# Blunt trauma causing thrombotic occlusive myocardial infarction

**DOI:** 10.21542/gcsp.2025.44

**Published:** 2025-08-30

**Authors:** Jace C. Bradshaw, AlleaBelle Bradshaw, Rishab Agarwal, P Logan Weygandt

**Affiliations:** 1 Department of Emergency Medicine and Anesthesiology and Critical Care, Johns Hopkins University School of Medicine; 2 Division of Cardiac Surgery, Department of Surgery, Johns Hopkins University School of Medicine; 3 Eastern Virginia Medical School

## Abstract

**Introduction:** Traumatic myocardial infarction (TMI) is a rare but serious complication of blunt chest trauma, typically arising from coronary artery dissection, intramural hematoma, or myocardial contusion. Early recognition and intervention are critical, but diagnosis can be challenging given the broad differential for chest pain in trauma patients.

**Case presentation:** A 66-year-old female presented to a quaternary academic emergency department after a motor vehicle collision with progressive chest pain. Initial electrocardiogram (ECG) showed hyperacute T-waves in lead III, ST depression with T-wave inversion in aVL, and ST depression in V2, with posterior leads revealing ST elevation in V7–V9. Trauma imaging ruled out aortic injury but revealed right coronary artery (RCA) occlusion. Left heart catheterization demonstrated complete occlusion of the mid-RCA, managed successfully with drug-eluting stent placement. The patient was discharged chest-pain free on hospital day four with plans for cardiac rehabilitation.

**Discussion:** This case highlights a rare presentation of TMI in an older patient, with RCA involvement rather than the more common left anterior descending artery involvement. While TMI often occurs in patients under 45 and typically results from coronary dissection, the occlusion observed in this case is most consistent with intraluminal thrombosis, though this cannot be definitively determined. Diagnosing TMI requires maintaining a high index of suspicion, as symptoms may mimic myocardial contusion. Timely PCI is preferred over thrombolytics, given the potential for underlying coronary artery dissection.

**Conclusion:** TMI, though rare, must be considered in trauma patients with chest pain and ischemic ECG changes. Early ECG acquisition and imaging are essential. PCI and CABG are the most common primary interventions, while thrombolytics should generally be avoided. Continued research is needed to refine diagnostic and management strategies for this complex condition.

## Introduction

Traumatic myocardial infarction (TMI) is a rare but significant complication primarily arising from blunt chest trauma. Contemporary literature identifies various mechanisms and clinical manifestations of this condition, highlighting its diverse presentations and the necessity for timely intervention. The mechanisms behind TMI generally include coronary artery dissections, intramural hematomas, and myocardial contusions resulting from blunt force impact. This report emphasizes the importance of accurate diagnosis of cardiovascular pathology in the trauma patient, including the role of radiological imaging in differentiating causes of chest pain in blunt trauma.

**Figure 1.**
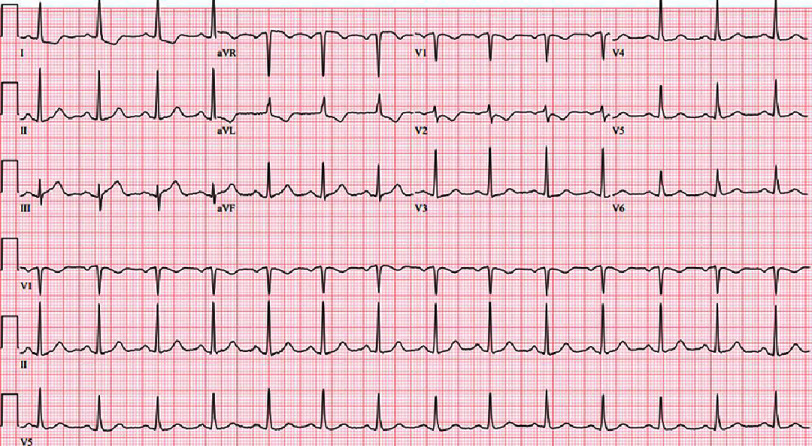
The ECG obtained on presentation to the emergency department. There are ST elevations in avR, borderline elevations in III, and with ST depressions in I, V2, and V3.

## Case report

A 66-year-old female with a history of hypertension and colon cancer presented to the emergency department (ED) one hour after a motor vehicle collision (MVC) in which she was an unrestrained rear-seat passenger. She had initially declined ambulance transport from the scene; however, she presented to the ED due to worsening chest pain that began following the collision.

**Figure 2.**
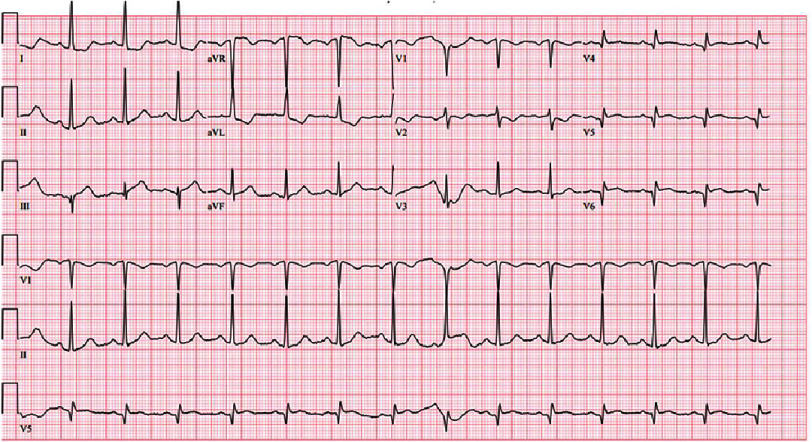
The posterior ECG 15 min after arrival the emergency department. The ST elevations in avR are more pronounced and the ST elevation in III appears to be increasing. There are ST depressions in I, V2, and V3. Because this is a posterior ECG, V4–6 represent V7–9 and show one mm ST elevation in lead V8.

**Figure 3.**
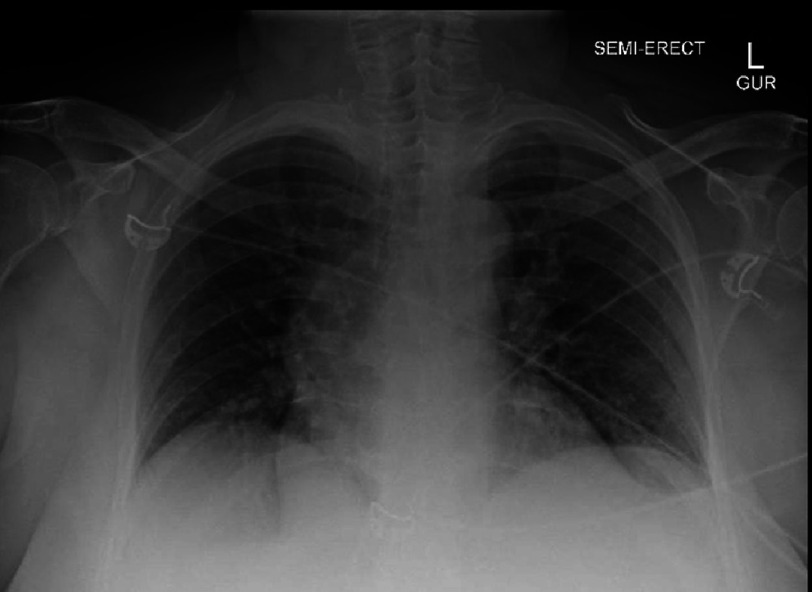
The chest X-ray showing a widened mediastinum.

**Figure 4.**
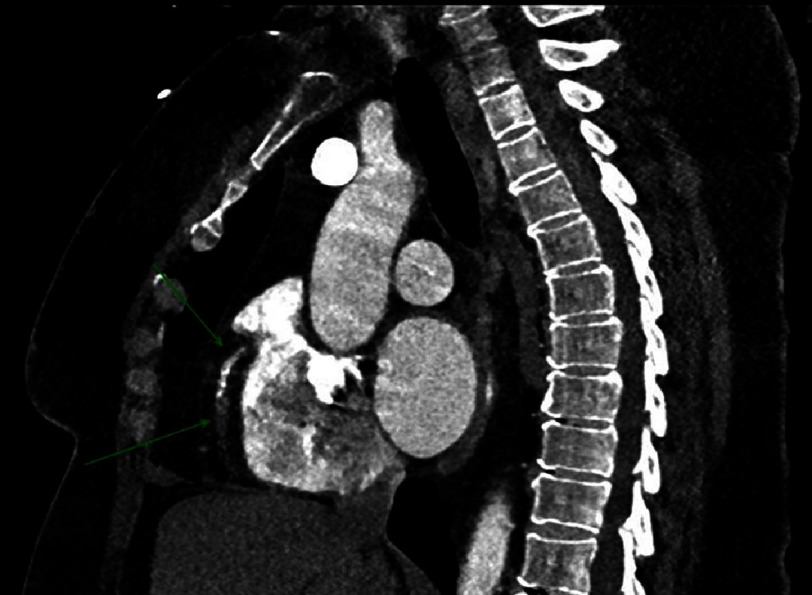
The CT chest with arterial contrast timed for dissection evaluation. The green arrows point to the RCA that appears to be occluded.

On arrival to our academic ED, the patient reported burning, left chest pain without radiation with associated dyspnea and nausea that she attributed to airbag deployment. An immediate electrocardiogram (ECG) demonstrated hyperacute T-waves in lead III, ST depression with T-wave inversion in aVL, and ST depression in V2 (Figure 1). This prompted a posterior ECG that showed 1–2 mm ST elevation in leads V7–V9 (Figure 2). Our “heart attack team” was activated for emergency left heart catheterization. Because the patient presented after a traumatic event, Advanced Trauma Life Support (ATLS) was continued. The primary survey initially revealed no injuries, but a chest X-ray obtained as an adjunct to the primary survey demonstrated mediastinal enlargement, raising concern for traumatic aortic dissection involving the coronaries (Figure 3). The patient was taken for computed tomography angiography (CTA) that did not show dissection or other traumatic injuries. However, the CTA did demonstrate a right coronary artery (RCA) occlusion (Figure 4). The patient’s initial troponin was 49 ng/dL and they had no other significant lab abnormalities. The one-hour repeat troponin would return 18,954 ng/dL after the patient left the emergency department.

**Figure 5.**
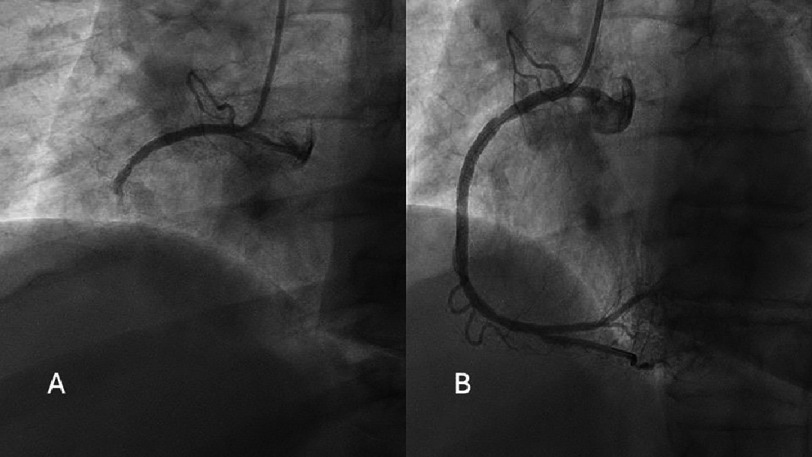
The left heart catheterization showing the occlusion with TIMI 0 flow (A) and post intervention with TIMI III flow (B) images.

Based on the ECG, the patient went to the catheterization suite and was found to have right dominant circulation and a completely occluded mid-RCA with TIMI-0 flow distally (Figure 5A). After drug-eluting stent deployment with a 3.5 × 38 mm Medtronic Onyx Frontier stent and dilation there was no residual stenosis and TIMI-3 flow restoration (Figure 5B). A post-procedural echocardiogram showed apical and septal hypokinesis with a preserved ejection fraction. On hospital day four, the patient was discharged chest pain free with plans for cardiac rehabilitation.

**Figure 6.**
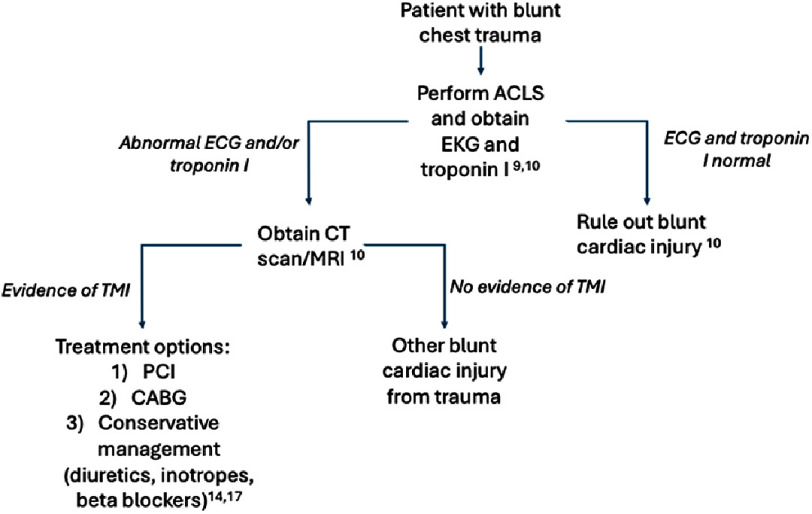
Clinical decision tree for TMI evaluation.

## Discussion

This case describes a TMI due to thrombosis of the RCA. Research suggests that TMIs are rare but are most described after severe blunt trauma. Motor vehicle accidents and other high-velocity impacts account for nearly 90% of cases^1,2^. Christensen et al. conducted a review of several cases of TMI, concluding that many of the cases described in the literature are due to dissection^3^. For example, Allemeersch et al. detail a case of traumatic coronary artery dissection leading to acute MI following blunt thoracic trauma, emphasizing the complex interplay between high energy trauma and coronary pathology^1^. Coronary artery dissection due to an intimal tear can cause occlusion as blood pools between layers of the artery wall, forming a hematoma. Neither CTA nor coronary catheterization showed evidence of coronary artery dissection in our patient, suggesting the TMI was secondary to intraluminal thrombosis. Beyond dissection, other mechanisms of occlusion in TMI include intraluminal thrombosis secondary to dislodgment of a plaque, vascular spasm, or external compression^3–5^. While the clinical presentation and angiographic findings in this case suggest intraluminal thrombosis, it is not possible to definitively determine the exact pathophysiological mechanism without advanced imaging or histopathological confirmation.

The age distribution described shows TMI occurs in younger patients compared to those with non-traumatic acute coronary syndrome with 82% of the patients with TMI after blunt chest trauma less than 45 years old. Interestingly, our patient’s demographic (60+ years old) comprised only 2.5% of patients^3^. Furthermore, the patient presented with an RCA occlusion resulting in inferior TMI. For comparison, Christensen et al. found that the left anterior descending artery (LAD) was most frequently affected (71.4%), followed by the RCA (19.0%), left main trunk (6.4%), and left circumflex artery (3.2%)^3^. This trend continues as more recent case reports describing TMI in blunt trauma describe an LAD lesion in the younger population as well^6–8^. In our case, diagnosing an older patient with an RCA occlusion is relatively uncommon among TMI presentations.

The differential of consequence for chest pain in trauma patients is broad: cardiac contusion, hemothorax, dissection of the great vessels, TMI, pericardial effusion or tamponade, sternal or rib fractures, pleural contusion, pneumothorax, esophageal injury, and referred or psychogenic pain. Therefore, a thorough and comprehensive approach to the history and physical exam is essential (Figure 6). The initial evaluation of a suspected acute coronary syndrome in the hospital setting should involve obtaining and interpreting an ECG within 10 min of presentation^9^. Furthermore, when blunt cardiac injury is suspected, both an ECG and troponin I levels should be obtained, according to the *Eastern Association for the Surgery of Trauma practice guideline*^10^. This is further supported by a 2023 systematic review and meta-analysis that found ECG and troponin I testing resulted in the highest sensitivity (85%) in ruling out myocardial injury in cases of blunt trauma, compared to other diagnostic tests (creatine phosphokinase-MB, echocardiography, troponin T)^11^. In this case report, the patient’s dramatic rise in troponin (49 to 18,954 ng/dL) is of clinical significance, as an increase of this magnitude correlates with significant myocardial injury^12^.

The presentation of TMI is further complicated by variance in clinical presentation, as patients may initially present with nonspecific symptoms that resemble those of myocardial contusion. Guo et al. describe a case in which a baseball impact resulted in syncope due to acute MI, illustrating the potential for syncope due to ventricular arrhythmias during critical phases of the cardiac cycle^13^. This variable presentation means that a high degree of clinical suspicion is warranted to overcome triage bias and achieve timely intervention. Therefore, TMI should always be suspected in patients with significant blunt chest trauma, until proven otherwise.

Like all myocardial infarctions, timely intervention is crucial in effectively managing TMI. Clinicians should be extremely cautious using thrombolytic therapy in this population because TMI is most commonly the result of dissection, meaning thrombolytic therapy might worsen the situation. After examining 179 cases of myocardial infarction secondary to blunt chest trauma, Marroush et al. determined that percutaneous coronary intervention (PCI) and coronary artery bypass grafting (CABG) are the most common initial therapies^14^. There is increasing preference for PCI in these cases, with reported high success rates when procedures are carried out by experienced interventionalists^15^. However, managing TMI via PCI can be challenging. In some instances, urgent CABG is necessary due to stent thrombosis following initial PCI, emphasizing the complexity of these cases^2^. Such complexity is compounded when patients also have extensive vascular involvement, which often necessitates meticulous planning and execution of interventional approaches to mitigate risks of adverse outcomes^16^. Interestingly, a report of TMI has described that conservative management, including diuretics, beta blockers, and cardiac stimulants is also an option if distal blood flow is normal despite proximal coronary artery pathology^17^. This highlights the unique management of TMI depending on the case.

To our knowledge, no study has reviewed long-term outcomes of patients with successful management of TMI from blunt force trauma, likely due to the rarity of this condition. Generally speaking, participation in cardiac rehabilitation has been associated with reduced readmission (Hazard ratio (HR) = 0.75, 95% confidence interval (CI) [0.65–0.87], *p* < 0.001) and mortality (HR = 0.58, 95% CI [0.49–0.68], *p* < 0.001) after myocardial infarction^18^. However, unique aspects to TMI including psychological stress and co-existing trauma may limit the generalizability of these findings.

### What have we learned?

The literature reflects a growing recognition of TMI as a serious clinical entity, demanding awareness of its etiology, potential complications, and evidence-informed management strategies. Compared to atraumatic MI, TMI occurs more commonly in younger patients and is most commonly caused by coronary dissection. Due to its variable presentation, TMI should always be suspected in patients with significant blunt chest trauma, until proven otherwise. Prioritizing ECG acquisition despite a history of traumatic injury is essential to rapidly identifying TMI. Treatment options include PCI and CABG while thrombolytic therapy should be avoided. Successful management requires effective communication amongst various disciplines including emergency medicine and cardiology. There is a need for ongoing research into the mechanisms, optimal interventions, and outcomes associated with TMI to enhance patient care and clinical outcomes in these complex scenarios.
